# Loss of Bile Salt Export Pump (Bsep/Abcb11) Ameliorates Toxin-induced Hepatic Fibrosis via Suppression of Hepatocellular Jun Amino-terminal Kinase Signaling and Hepatic Stellate Cell Activation

**DOI:** 10.1016/j.jcmgh.2025.101630

**Published:** 2025-09-12

**Authors:** Claudia D. Fuchs, Emmanuel D. Dixon, Philipp Königshofer, Thierry Claudel, Veronika Mlitz, Hubert Scharnagl, Tatjana Stojakovic, Thomas Reiberger, Michael Trauner

**Affiliations:** 1 Hans Popper Laboratory of Molecular Hepatology, Division of Gastroenterology and Hepatology, Department of Medicine III, Medical University of Vienna, Vienna, Austria; 2 Experimental Hepatic Hemodynamic lab (HEPEX), Division of Gastroenterology and Hepatology, Department of Medicine III, Medical University of Vienna, Vienna, Austria; 3 Christian Doppler laboratory for Portal Hypertension and Liver Fibrosis, Medical University of Vienna, Vienna, Austria; 4 CeMM Research Center for Molecular Medicine of the Austrian Academy of Sciences, Vienna, Austria; 5 Clinical Institute of Medical and Chemical Laboratory Diagnostics, Medical University of Graz, Graz, Austria; 6 Clinical Institute of Medical and Chemical Laboratory Diagnostics, University Hospital Graz, Graz, Austria

**Keywords:** Fibrosis, Hydroxylated bile acids, Hepatic stellate cells

## Abstract

**Background & Aims:**

Loss of Bsep/Abcb11 results in a hydrophilic bile acid (BA) pool consisting of tetrahydroxylated BAs (THBAs) reducing cholestasis-induced liver injury. In this study, we investigated whether loss of Bsep may protect mice from development of toxin-induced liver fibrosis by directly impacting on hepatic stellate cell (HSC) activation.

**Methods:**

Wild-type (WT) and *Bsep*^*-/-*^ mice were exposed to carbon tetrachloride (CCl_4_) or thioacetamide (TAA) for 4 weeks (3 injections per week) as models of toxin-induced liver fibrosis. In vitro, the human HSC line LX2 and immortalized human hepatocytes (IHHs) were challenged with TGFβ or 12S-HETE (arachonidonic acid derivate) with or without THBA treatment. Liver immunohistochemistry (IHC), immunofluorescence (IF), gene and protein expression, intrahepatic BA profile, luciferase activity, and 12S-HETE assays were performed.

**Results:**

In contrast to WT mice, serum transaminases were not elevated in *Bsep*^*-/-*^ mice after CCl_4_ or TAA injection. Accordingly, IHC accompanied by gene expression profiling and measurement of hepatic hydroxyproline levels reduced hepatic inflammation and fibrosis in *Bsep*^*-/-*^ mice challenged with CCl_4_ or TAA. Mechanistically, hepatic protein expression of pJNK (a known mediator of CCl_4_-induced liver fibrosis) was reduced in *Bsep*^*-/-*^ CCl_4_ mice in comparison to CCl_4_-exposed WT mice. In vitro, activation of JNK was suppressed by THBA in IHH cells. LX2 cell activation was attenuated by treatment with THBA as reflected by reduced AP1-luciferase activity and by restoring gene expression levels of p62 and Nrf2 as well as by significant reduction of *αSma, Tgfβ,* and *Mcp-1* gene expression.

**Conclusions:**

Loss of Bsep protects mice from toxin-induced liver fibrosis via suppression of hepatocellular pJNK signaling and attenuation of HSC activation.

## Graphical abstract

**Figure:**
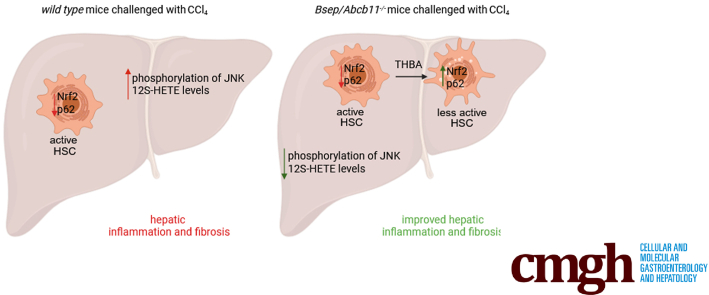

**Summary**

Absence of Bsep/Abcb11, concomitant with the presence of tetrahydroxylated bile acids, protects mice from toxin induced hepatic fibrosis via suppression of hepatocellular jun amino-terminal kinase phosphorylation. Furthermore, tetrahydroxylated bile acids have a direct anti-fibrotic effect on hepatic stellate cells via restoring p62 and Nrf2 signaling.

Chronic unresolved liver injuries of different etiologies lead to an imbalance between production and dissolution of extracellular matrix (ECM). Hepatic stellate cells (HSCs) (shown to be the primary source of ECM) act in concert with hepatocytes and immune cells to aggravate scarring in response to liver injury.^1^ Apoptotic^2^^,^^3^ and stressed^4^ hepatocytes provoke inflammatory cell recruitment to the liver and the release of proinflammatory as well as pro-fibrotic cytokines such as transforming growth factor beta (TGFβ). Liver fibrosis may evolve further into cirrhosis and eventually hepatocellular carcinoma. To date, there is no specific therapy for patients suffering from hepatic fibrosis, and liver transplantation is often necessary when patients progress to cirrhosis portal hypertension and liver failure.^5^

Strategies that target bile acid (BA) signaling hold the potential for treatment of patients with liver disease.^6^, ^7^, ^8^ In addition to their anti-cholestatic effects,^9^ therapeutic BAs such ursodeoxycholic acid (UDCA) and its side chain shortened derivative norucholic acid (NCA, formerly norUDCA) were shown to be beneficial in thioacetamide (TAA)-induced fibrosis.^10^ Furthermore, NCA was shown to be protective against liver fibrosis provoked by *Schistosoma mansoni* infection.^11^ This observation was also recently extended to tetrahydroxylated bile acids (THBAs),^12^ which are very hydrophilic and thus potentially less toxic BA species formed in mice lacking bile salt export pump (Bsep/Abc11) and protecting these mice from (further) acquired cholestatic liver injury.^13^^,^^14^ *Bsep*^*-/-*^ animals have increased expression of Cyp3a11 and Cyp2b10,^14^ 2 enzymes being key for BA hydroxylation.^14^ Hydroxylated BAs are secreted via alternative BA transporters Mrp3 and Mrp4 (both increased in *Bsep*^*-/-*^ animals^14^) into the blood and excreted via urine. Moreover, THBAs were shown to reduce cytokine secretion from hepatocytes due to suppression of early growth response 1 (Egr1), a critical driver of hepatic inflammation under cholestatic conditions. Thereby, THBA attenuated the numbers of infiltrating neutrophils and macrophages in livers of *Mdr2*^*-/-*^ mice as well as macrophage activation in vitro,^13^ indicating immunomodulatory effects of this BA, which may result in attenuation of hepatic fibrosis development.

In the present study, we aimed to investigate the hypothesis that THBAs may have a protective role in development of hepatic fibrosis by directly interfering with pathways involved in development of hepatic fibrosis and/or inhibiting activation of HSCs. Therefore, *Bsep*^*-/-*^ mice (with THBAs as one of the most prominent BA species in their BA pool^13^^,^^14^) were subjected to carbon tetrachloride (CCl_4_) and TAA treatment, commonly used to induce of toxin-mediated liver fibrosis.^15^

## Results

### Absence of Bsep Prevents Toxin-induced Liver Injury

**Figure 1:**
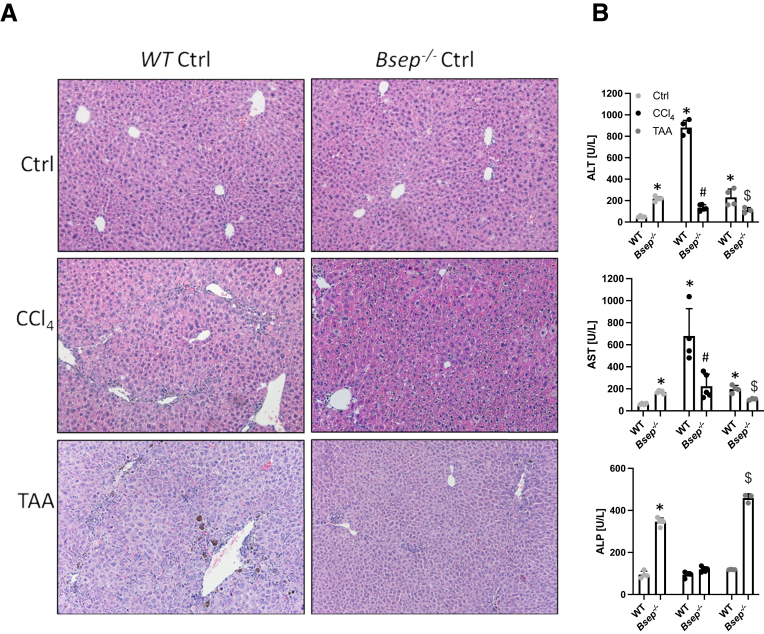
**Loss of Bsep improves CCl_4_-induced liver injury.** (*A*) Representative H&E images (×10 magnification). (*B*) Serum biochemistry reflects reduced levels of transaminases (ALT, AST) in *Bsep*^*-/-*^ mice subjected to CCl_4_ or TAA treatment compared with WT mice. ALP levels remained unchanged due to CCl_4_ challenge in WT mice and decreased in *Bsep*^*-/-*^ mice. TAA challenge led to increase of ALP levels in *Bsep*^*-/-*^ mice. ∗Significant difference from WT mice; ^#^significant difference from WT CCl_4_ mice; ^$^significant difference from WT TAA mice; *P* < .05.

After 4 weeks of CCl_4_ administration, *Bsep*^*-/-*^ mice did not show histological features of liver injury (Figure 1*A*), whereas in wild-type (WT) mice challenged with CCl_4_, pronounced histological features of hepatic inflammation and fibrosis were present (Figure 1*A*). Furthermore, serum levels of alanine aminotransferase (ALT) and aspartate aminotransferase (AST) as markers of hepatocellular injury remained normal in CCl_4_-subjected *Bsep*^*-/-*^ mice but were profoundly elevated in CCl_4_-injected WT mice (Figure 1*B*). Although CCl_4_ in both WT and *Bsep*^*-/-*^ mice did not influence levels of alkaline phosphatase (ALP), serum BA concentration increased in WT mice after CCl_4_ application (Figure 1*B*). Of note, *Bsep*^*-/-*^ mice were also protected from TAA-induced liver injury reflected by improved liver transaminases (Figure 1*A and B*). However, liver injury was more severe in WT animals subjected to CCl_4_ than in the TAA setting (Figure 1*B*). Notably, intrahepatic BA profiling revealed that challenging *Bsep*^*-/-*^ mice with CCl_4_ led to a 40% reduction of THBA levels; TAA application even reduced intrahepatic THBA levels by 80% when compared with untreated *Bsep*^*-/-*^ mice (Table 1).

**Table 1:** Intrahepatic BA Profiling

pmol/mg	WT Ctrl	*Bsep*^*-/-*^ Ctrl	WT CCl_4_	*Bsep*^*-/-*^ CCl_4_	WT TAA	*Bsep*^*-/-*^ TAA
TCA	11.72 ± 7.90	18.92 ± 3.91	24.72 ± 14.07	21.06 ± 7.44	22.91 ± 9.81	30.38 ± 4.98^b^^,^^c^
TUDCA	0.17 ± 0.09	0.7 ± 0.14	0.26 ± 0.2	0.49 ± 0.11	0.39 ± 0.21	0.53 ± 0.07
TCDCA	0.29 ± 0.14	0.6 ± 0.13	0.55 ± 0.68	0.41 ± 0.10	0.46 ± 0.25	0.67 ± 0.14
TDCA	1.11 ± 0.32	0.05 ± 0.04	0.88 ± 0.39	0.04 ± 0.03	0.8 ± 0.44	0.09 ± 0.02
ToMCA	2.13 ± 0.89	10.97 ± 3.17^a^	2.93 ± 1.38	9.22 ± 3.92	5.43 ± 2.93^a^	28.22 ± 4.41^b^^,^^c^
TbMCA	4.1 ± 1.96	85.44 ± 19.54^a^	14.76 ± 10.77	115 ± 38.66	11.31 ± 4.57^a^	138.42 ± 27.13^c^
CA	0.45 ± 0.36	0.02 ± 0.01	0.85 ± 1.05	0.04 ± 0.01	1.12 ± 0.82	0.07 ± 0.05
oMCA	0.08 ± 0.04	0.17 ± 0.03	0.07 ± 0.04	0.21 ± 0.03	0.4 ± 0.28	0.58 ± 0.29
bMCA	0.03 ± 0.02	2.85 ± 0.63^a^	0.31 ± 0.18	4.87 ± 1.21	1.04 ± 0.82	3.98 ± 1.83
TTHBA	n.d	49.19 ± 17.88	0.7 ± 0.53	20.92 ± 3.33^a^	0.34 ± 0.21	10.95 ± 4.76^b^^,^^c^
TPHBA	n.d	0.44 ± 0.15	n.d	0.14 ± 0.03	n.d	0.06 ± 0.04
Summ	20.9 ± 11.4	169.35 ± 23.76^a^	46.03 ± 28.51	172.46 ± 37.29	44.71 ± 18.78	214.16 ± 42.89^b^^,^^c^

BA, bile acid; bMCA, betamuricholic acid; CA, cholic acid; CCl_4_, carbon tetrachloride; Ctrl, control; oMCA, omegamuricholic acid; TAA, thioacetamide; TbMCA, taurobetamuricholic acid; TCA, taurochenodeoxycholic acid; TCDCA, taurochenodeoxycholic acid; TDCA, taurodeoxycholic acid, ToMCA, tauroomegamuricholic acid; TPHBA, tauropentahydroxylated bile acid; TTHBA, taurotetrahydroxylated bile acid; TUDCA, tauroursodeoxycholic acid; WT, wild-type.

a Significant difference from WT mice; *P* < .05.

b Significant difference from *Bsep*^*-/-*^ mice; *P* < .05.

c Significant difference from *Bsep*^*-/-*^ CCl_4_ mice; *P* < .05.

### Absence of Bsep Protects From CCl_4_-induced Liver Inflammation and Fibrosis

**Figure 2:**
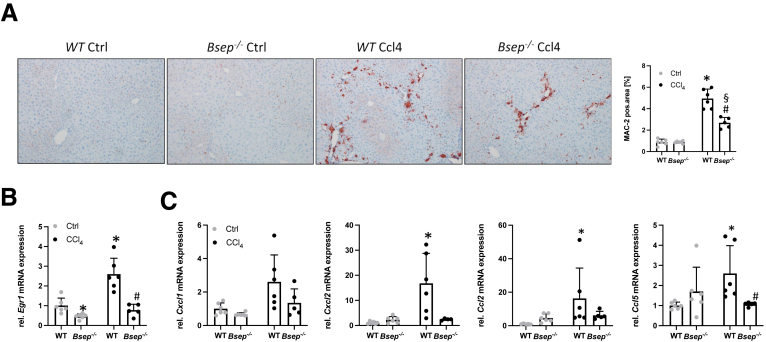
**Loss of Bsep improves CCl_4_-induced liver inflammation.** (*A*) Representative MAC-2/Galectin3 IHC images (×10 magnification) as well as computational analysis show reduced numbers of macrophages in the livers of *Bsep*^*-/-*^ mice. Real-time PCR was used to assess the mRNA expression of (*B*) *Egr1* and its downstream targets (*C*) *Cxcl1, Cxcl2, Ccl2,* and *Ccl5* as markers of inflammation. All of them were reduced in *Bsep*^*-/-*^ mice subjected to CCl_4_ treatment compared with WT mice. mRNA expression values were normalized against 36b4 levels and are shown relative to expression level in WT controls. ∗Significant difference from WT mice; ^#^significant difference from WT CCl_4_ mice; ^§^significant difference from *Bsep*^*-/-*^ mice; *P* < .05. Computational analysis of histological pictures was done via image J 1.51j8.

**Figure 3:**
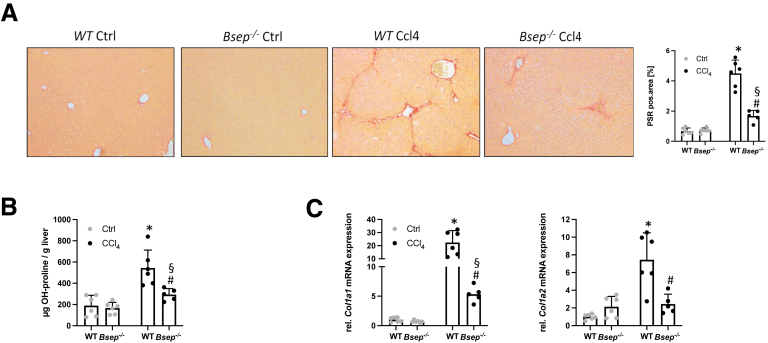
**Loss of Bsep improves CCl_4_-induced liver fibrosis.** (*A*) Representative PSR stainings (×10 magnification) as well as computational analysis show improved hepatic fibrosis in *Bsep*^*-/-*^ mice. (*B*) OH-proline content was reduced in *Bsep*^*-/-*^ mice subjected to CCl_4_ compared with challenged WT mice. (*C*) Real-time PCR was used to assess the mRNA expression of fibrotic marker collagen type I alpha 1 (Col1a1) and Col1a2, which were reduced in *Bsep*^*-/-*^ mice subjected to CCl_4_. mRNA expression values were normalized against 36b4 levels and are shown relative to expression level in WT controls. ∗Significant difference from WT mice; ^#^significant difference from WT CCl_4_ mice; ^§^significant difference from *Bsep*^*-/-*^ mice; *P* < .05. Computational analysis of histological pictures was done via image J 1.51j8.

Next, we investigated markers of hepatic inflammation (Figure 2) and fibrosis (Figure 3) in more detail at the mRNA as well as protein level. Representative images of MAC-2/Galectin-3 immunohistochemistry (IHC) as well as computational analysis showed significantly less amounts of MAC-2 positive cells in liver of *Bsep*^*-/-*^ mice subjected to CCl_4_ in comparison to challenged WT animals (Figure 2*A*). mRNA expression of *Egr1* (a key driver of hepatic inflammation, and known to be induced via CCl_4_^16^) was increased in WT CCl_4_-challenged mice and remained unchanged in *Bsep*^*-/-*^ mice upon CCl_4_ challenge (Figure 2*B*). Accordingly, gene expression profiling of *Egr1* downstream targets such as *Cxcl1, Cxcl2, Ccl2,* and *Ccl5* revealed a profound induction in WT mice upon CCl_4_ injection, whereas in CCl_4_-challenged *Bsep*^*-/-*^ mice, these markers remained at baseline levels, similar to unchallenged control mice (Figure 2*C*).

**Figure 4:**
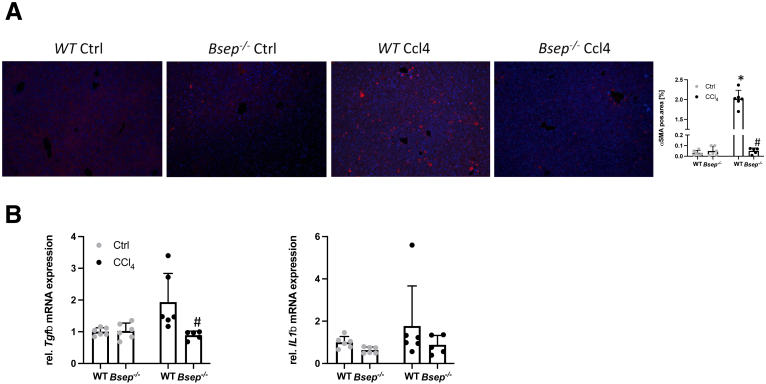
**Loss of Bsep improves CCl_4_-induced activation of HSCs.** (*A*) IF staining and computational analysis show reduced numbers of αSMA-positive cells in livers of *Bsep*^*-/-*^ mice subjected to CCl_4_ treatment compared with challenged WT mice. (*B*) Real-time PCR was used to assess the mRNA expression of markers of activated HSCs *Tgfβ* and *IL1β*, which were reduced in *Bsep*^*-/-*^ mice subjected to CCl_4_. mRNA expression values were normalized against 36b4 levels and are shown relative to expression level in WT controls. ∗Significant difference from WT mice; ^#^significant difference from WT CCl_4_ mice; *P* < .05. Computational analysis of histological pictures was done via image J 1.51j8.

**Figure 5:**
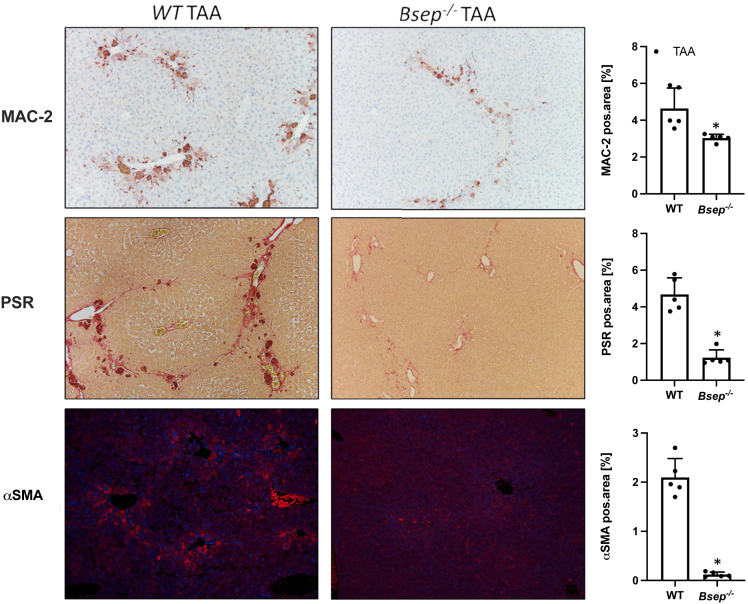
**Loss of Bsep improves TAA induced liver injury.** Representative MAC-2/Galectin3 IHC images (×10 magnification) as well as computational analysis show reduced numbers of macrophages in the livers of *Bsep*^*-/-*^ mice. Representative PSR stainings (×10 magnification) as well as computational analysis show improved hepatic fibrosis in *Bsep*^*-/-*^ mice. IF staining and computational analysis show reduced numbers of αSMA-positive cells in livers of *Bsep*^*-/-*^ mice. ∗Significant difference from WT TAA mice; *P* < .05.

Representative Picro-Sirius red (PSR) images as well as computational analysis displayed significantly lower collagen staining in *Bsep*^*-/-*^ animals after CCl_4_ application compared with challenged WT mice (Figure 3*A*). In addition, intrahepatic levels of hydroxyproline (OH-proline) were significantly lower in *Bsep*^*-/-*^ mice exposed to CCl_4_ in comparison with WT CCl_4_ animals (Figure 3*B*). In line, mRNA expression of fibrotic markers *Col1a1* and *Col1a2* was significantly lower in *Bsep*^*-/-*^ CCl_4_-injected mice compared with challenged WT mice (Figure 3*C*). In accordance with reduced hepatic fibrosis in *Bsep*^*-/-*^ CCl_4_-injected mice, numbers of alpha smooth muscle actin (αSMA)-positive cells (marker for activated HSCs), remained negligable when compared with WT CCl_4_-injected mice (Figure 4*A*). Subsequently, mRNA expression profiles of proinflammatory and profibrogenic cytokines such as *Tgfβ* and interleukin 1β (*IL1β*) known to be secreted from activated HSCs^17^^,^^18^ tended to be higher in WT mice upon CCl_4_ challenge (Figure 4*B*). Of note, *Bsep*^*-/-*^ animals challenged with TAA showed also reduced hepatic inflammation (MAC-2) and hepatic fibrosis (PSR) as well as reduced numbers of αSMA-positive cells when compared with WT TAA animals (Figure 5).

### Lack of Bsep Attenuates CCl_4_-induced Phosphorylation of Jun Amino-terminal Kinases

**Figure 6:**
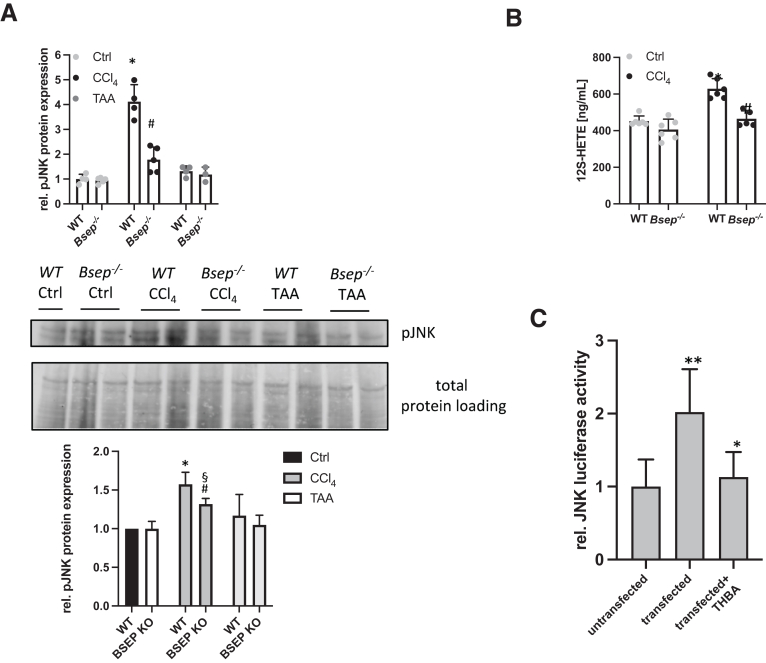
**Loss of Bsep attenuates phosphorylation of JNK in vivo.** Primary hepatocytes were isolated and (*A*) pJNK ELISA revealed increased phosphorylation of JNK in WT mice exposed to CCl_4_, whereas in *Bsep*^*-/-*^ mice pJNK levels remained unchanged. TAA exposure did not influence pJNK levels in WT and *Bsep*^*-/-*^ mice. Data are shown relative to WT Ctrl group. Western blot from primary hepatocytes revealed that loss of Bsep result in lower levels of JNK phosphorylation in the presence of CCl_4_. TAA exposure did not impact on pJNK levels. Western blot was quantified using ImageLab 10.0. ∗Significant difference from WT mice; ^#^significant difference from WT CCl_4_ mice; ^§^significant difference from *Bsep*^*-/-*^ Ctrl mice; *P* < .05. (*B*) 12 (S)-HETE ELISA showed reduced concentration of 12 (S)-HETE in serum of *Bsep*^*-/-*^ mice subjected to CCl_4_ compared with treated WT mice. ∗Significant difference from WT mice; ^#^significant difference from WT CCl_4_ mice; *P* < .05. (*C*) Human IHHs were transfected with a JNK-luciferase construct and treated with THBA. THBA attenuated JNK activation. ∗∗Significant difference from untransfected cells; ∗significant difference from transfected cells; *P* < .05.

Because CCl_4_ induces phosphorylation of jun amino-terminal kinases (JNK), leading to activation of the arachidonic acid signaling cascade, resulting in production of the proinflammatory trigger 12-hydroxyeicosatetraenoic acid (12S-HETE),^19^ we next investigated whether this key fibrogenic/pro-inflammatory mechanism is altered in *Bsep*^*-/-*^ mice. pJNK enzyme-linked immunosorbent assay (ELISA), and quantification of pJNK immune blot revealed that phosphorylation of JNK was induced upon CCl_4_ challenge in WT animals but not in *Bsep*^*-/-*^ mice (Figure 6*A*). Subsequently, serum concentrations of 12S-HETE were significantly lower in *Bsep*^*-/-*^ mice upon CCl_4_ injection when compared with challenged WT mice (Figure 6*B*). (TAA challenge did not interfere with levels of JNK phosphorylation, neither in WT nor in *Bsep*^*-/-*^ mice [Figure 6*A*]). Of note, in immortalized human hepatocytes (IHHs) transfected with the JNK-luciferase reporter, JNK activity was significantly reduced by treatment with THBA (Figure 6*C*), indicating that THBAs, known to be present in *Bsep*^*-/-*^ mice,^13^^,^^14^ may counteract the toxin-induced JNK signaling cascade, thus protecting from development of hepatic inflammation and fibrosis.

### THBA Counteracts HSC Activation In Vitro

**Figure 7:**
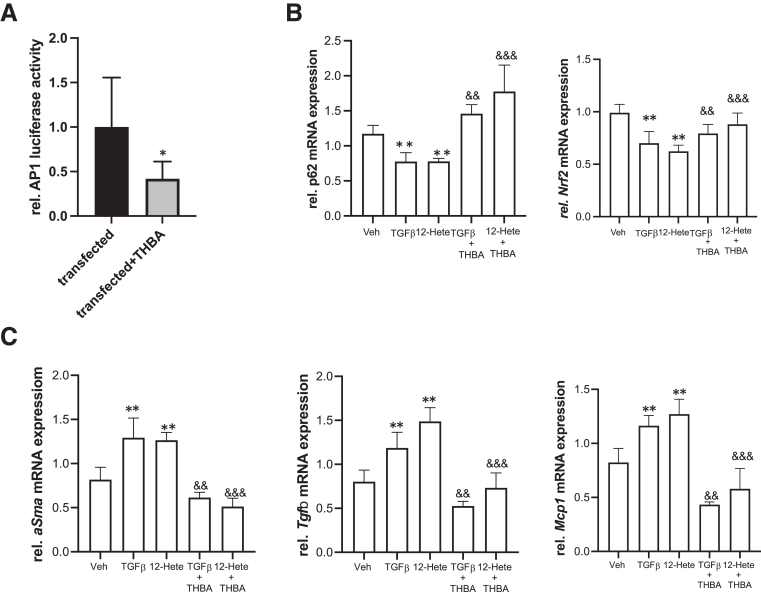
**THBA improves TGFβ and 12-HETE-induced activation of HSCs, in vitro.** (*A*) The human HSC line LX2 was transfected with an AP1-luciferase construct and treated with THBA. THBA attenuated AP1activation. (*B and C*) LX2 cells were treated either with TGFβ or with 12S-HETE or with a combination of TGFβ and THBA or 12S-HETE and THBA. (*B*) Gene expression profiling showed that THBA restored *p62* and *Nrf2* expression, which was reduced due to TGFβ and 12S-HETE treatment. (*C*) mRNA expression of markers for activated HSCs, *αSma*, *Tgfβ,* and *Mcp1* and were reduced in HSCs treated with TGFβ and THBA or 12S-HETE and THBA compared with mono-treatment of TGFβ and 12S-HETE. mRNA expression values were normalized against 36b4 levels and are shown relative to expression level in untreated controls. ∗∗Significant difference from control cells; ∗significant difference from CCl_4_-treated cells; ^&&^significant difference from TGFβ-treated cells; ^&&&^significant difference from 12S-HETE-treated cells; *P* < .05.

Even though intrahepatic THBA levels were reduced due to CCl_4_ and TAA intoxication, in vitro, we investigated whether THBAs may have anti-fibrotic properties and thereby protecting *Bsep*^*-/-*^ mice from toxin-induced fibrosis. Therefore, LX2 cells were transfected with the AP1-luciferase reporter. AP1 activation, also contributing to activation of HSCs,^20^ was significantly reduced due to THBA treatment (Figure 7*A*).

Furthermore, the LX2 cells were treated either with TGFβ or 12S-HETE (as a proinflammatory metabolite produced via the JNK signaling cascade) as potential activators of HSCs as well as with combinations either of TGFβ/THBA or 12S-HETE/THBA (Figure 7*B and C*). Although both TGFβ and 12S-HETE resulted in reduced expression of *p62* and *Nrf2* (known to have antioxidative as well as antifibrotic properties^21^), cotreatment with THBA restored expression levels of both markers (Figure 7*B*). Accordingly, THBA was able to significantly reduce TGFβ or 12S-HETE induced gene expression of profibrotic markers such as *αSma, Tgfβ,* and *Mcp1* (Figure 7*C*). Together, these findings support our hypothesis that THBAs, as one of the predominant BAs in *Bsep*^*-/-*^ mice, protect them from toxin-induced liver injury by direct antifibrotic mechanisms.

## Discussion

The present study demonstrates that mice lacking the canalicular BA export pump Bsep/Abcb11 are protected from toxin-induced liver fibrosis independent from potential anti-cholestatic actions. Mechanistically, we were able to show that pJNK signaling, known to be a key pathway in CCl_4_-induced liver injury,^19^^,^^22^ was repressed in *Bsep*^*-/-*^ mice with a hydrophilic BA pool. In line, THBA, a prominent BA species in *Bsep*^*-/-*^ mice,^13^^,^^14^ attenuated JNK signaling in human hepatocytes and proinflammatory/profibrogenic pathways in HSCs via restoring *p62* and *Nrf2* expression.

The hydrophobic BA taurocholic acid (TCA) was previously reported to directly activate phosphorylation of JNK.^22^ Because in *Bsep*^*-/-*^ mice the relative contribution of TCA to the total BA pool is rather negligible (^14^ and Table 1), this observation supports our finding of reduced hepatic JNK phosphorylation in *Bsep*^*-/-*^ mice. Given the predominance of THBA, a certain diluting effect attenuating proinflammatory effects of TCA (supposedly via JNK activation) may be involved in supporting hepatocecllular protection in *Bsep*^*-/-*^ mice. However, our in vitro findings in human hepatocytes also support a direct inhibitory effect of JNK phosphorylation via THBA as well.

Reduced phosphorylation/activation of JNK may also explain the low levels of 12S-HETE in serum of *Bsep*^*-/-*^ mice. Production of 12S-HETE, a member of the proinflammatory eicosanoids, secreted from immune cells as well as hepatocytes^23^ and responsible for recruitment of macrophages and neutrophils to the liver, is controlled by JNK activity.^19^ Our in vitro data support direct inhibitory effects of THBA on the pJNK/12S-HETE pathway.

Although EGR1 has been described as a stimulator of fibrogenesis,^24^^,^^25^ in the CCl_4_ model, it seems to play a hepatoprotective role during liver regeneration as a very early event in response to liver injury.^16^ In contrast to WT animals, in the *Bsep*^*-/-*^ mouse model Egr1 expression was not induced upon CCl_4_ challenge, which is in line with our previous findings that THBAs repress Egr1 expression.^13^ As such, in the *Bsep*^*-/-*^ CCl_4_ setting, Egr1 might not play a decisive role in counteracting development of hepatic fibrosis.

In the human HSC line LX2, THBA treatment reduced AP1-luciferase activity, restored p62 and Nrf2 expression, and ameliorated TGFβ and 12S-HETE-induced HSC activation, reflecting a possible direct anti-fibrotic effect of THBA. Lower levels of hepatic fibrosis found in CCl_4_- and TAA-injected *Bsep*^*-/-*^ mice might be a result of both direct antifibrotic and anti-inflammatory/immunomodulatory effects of THBA because proinflammatory cytokines and chemokines such as *Tnfβ, IL1β,* and *Tgfβ* secreted from activated macrophages, are known to activate HSCs.^26^

In conclusion, our results demonstrate that THBAs have direct anti-inflammatory as well as direct antifibrotic effects via counteracting the JNK signaling cascade and decreasing the production of cytotoxic metabolites such as 12S-HETE in hepatocytes. Moreover, they counteract HSC activation via restoring p62 and Nrf2 expression. Thus, future studies should evaluate if therapeutic strategies capable of increasing THBAs can ameliorate liver injury in patients with liver disease.

## Materials and Methods

### Animals

Male FVB/N WT and *Bsep*^*-/-*^ mice were kindly provided by the British Columbia Cancer Research Center^27^ and bred as reported previously.^13^^,^^14^^,^^28^^,^^29^ As described,^30^ age-matched 8-week-old male littermates were injected intraperitoneally either with CCl_4_ 1 mL/kg 25% solution in olive oil (Sigma-Aldrich) or 150 mg/kg TAA 3×/week for a time period of 4 weeks. From one batch of animals, primary hepatocytes were isolated after liver perfusion. For direct comparison, serum biochemistry, pJNK ELISA and pJNK Western blot were performed from this animal batch (n = 3–5). Histology, IHC/immunofluorescence (IF), RNA analysis, 12S-HETE assay and intrahepatic BA profile were performed from separate batches (n = 5–7). This animal study was approved by the Animal Ethics Committee of the Medical University of Vienna and the Federal Ministry of Science, Research, and Economy (BMWFW-66.009/008-WF/V/3b/2015) and was performed according to the Animal Research: Reporting of In Vivo Experiments (ARRIVE) guidelines.

### Serum Biochemistry and Histology

Serum biochemistry and histological staining (hematoxylin and eosin [H&E], PSR) was performed as described previously.^31^

### IHC/IF

Detection of hepatic MAC-2 and αSMA was performed as described previously.^32^^,^^33^ In brief, IHC for MAC-2/Galectin-3 or IF for αSMA-positive cells was performed on formaldehyde (4% neutral-buffered)-fixed, paraffin-embedded liver sections using monoclonal mouse antibodies. Sections were deparaffinated, rehydrated, and digested with 0.1% protease. Endogenous peroxidase was blocked with 1% H_2_O_2_ in methanol. Specific binding of the MAC-2 antibody was detected using a biotinylated anti-rat IgG and the ABC-System with amino-9-ethyl-carbazole as substrate. For immunofluorescence, slides were incubated with a monoclonal anti-mouse α-SMA antibody (ab5694, Abcam). Slides were then washed and incubated with the secondary antibody: Alexa Fluor 594 goat anti rabbit IgG (Thermo Fisher Scientific).

### Messenger RNA Analysis and Polymerase Chain Reaction

RNA isolation from liver, complementary DNA synthesis and real-time polymerase chain reaction (PCR) were performed as described previously.^34^ Oligonucleotide sequences are available upon request.

### Luciferase Activity Assay in IHHs

IHH^35^ cells were seeded in a 24-well plate and transiently transfected with 0.3 μg/well of an JNK-luciferase construct (a gift from Dr C. Glineur, Pasteur Institute) using Fugene transfection reagent (Promega) in sterile Opti-modifed Eagle’s medium for 12 hours. Cells were incubated in complete media containing 10 μM THBA (3α, 6α, 7α, 12α tetrahydroxycholanoic acid; UHN Shanghai Research & Development) for 24 hours. Cells were lysed using a solution of 4% Triton X-100, glycylglycine 100 mM, MgSO4100 mM, and ethylene glycol tetra acetic acid 250 mM for 1 hour at room temperature on a shaker platform. The obtained extracts were then combined with the solution containing the substrate (luciferin 2.5 mM and adenosine triphosphate 20 mM; Sigma-Aldrich) and analyzed by a luminometer (Lumat LB950; EG&G Berthold).

### Human HSC Culture

LX-2 cells, an immortalized HSC line,^36^ kindly provided by Prof S.L. Friedman (Mount Sinai School of Medicine) were cultured with Dulbecco’s modified Eagle’s medium (DMEM) (Life Technologies) supplemented with 5% non–heat-inactivated fetal bovine serum (FBS) and 1% penicillin/streptomycin solution (EuroClone). Upon reaching approximately 80% confluency, LX-2 cells were detached from the culture flask using 0.25% trypsin-EDTA solution and reseeded in 6 well plates at a density of 1 × 10^6^ cells/well. Cells were cultured in DMEM supplemented with 4 ng/mL TGFβ (Sigma-Aldrich), 10 μM THBA (3α, 6α, 7α, 12α tetrahydroxycholanoic acid; UHN Shanghai Research & Development), and 100 nM 12S-HETE (Sigma-Aldrich) for 24 hours. For transfection, LX-2 were seeded in a 24-well-plate and transiently transfected with 150 ng/well of an AP-1-luciferase (from Takara Bio) construct using Fugene transfection reagent (Promega) in sterile DMEM without FBS for 12 hours. Complete medium was then added for 48 hours with/without 100 μM of THBA and the cells lysed (lysis solution: 4% Triton-X100, Glycyl-Glycine 100 mM, MgSO4 100 mM, EGTA 250 mM) for 30 minutes at room temperature on a shaker. The extracts were then analyzed with a luminometer (Lumat LB9507 EG&G Berthold) injecting 100 μL of a solution (Luciferin 2.5 mM and ATP 20 mM, Sigma-Aldrich).

### Intrahepatic BA Analysis

Intrahepatic BA profiles were acquired using ultra-performance liquid chromatography tandem mass spectrometry as described previously.^37^

### Phospho-JNK ELISA

Total protein was isolated from primary hepatocytes and concentration was obtained using Pierce BCA Protein Assay Kit from ThermoFisher Scientific. 100 μg protein were used to conduct the RayBio Phospho-JNK (Thr183/Tyr185) ELISA (Catalog Number PEL-JNK-T183) according to manufacturer’s protocol. Optical density (OD) was measured at 450 nm (OD, 450/100 μg protein).

### Western Blotting

Total protein was isolated from primary hepatocytes, and concentration was obtained using Pierce BCA Protein Assay Kit from ThermoFisher Scientific. Target protein expression was normalized to total loaded protein amount, according to manufacturer’s instructions.

### 12S-HETE Assay

*12S-HETE* levels were measured in serum of animals according to the manufacturers’ manuals (Abcam; product number: ab133034).

### Statistical Analysis

Results were evaluated using GraphPad Prism 8.4.1. Statistical analysis was performed using 2-way analysis of variance (ANOVA). Data were reported as means of 5 to 7 animals per group ± standard deviation (SD). A *P* value ≤ .05 was considered significant.
