# Correction

**DOI:** 10.1016/j.jcmgh.2025.101708

**Published:** 2025-12-15

Fuchs CD, Dixon ED, Königshofer P, et al. Loss of Bile Salt Export Pump (Bsep/Abcb11) Ameliorates Toxin-induced Hepatic Fibrosis via Suppression of Hepatocellular Jun Amino-terminal Kinase Signaling and Hepatic Stellate Cell Activation. Cell Mol Gastroenterol Hepatol 2026;20:101630.

In the above article, the authors regret that two contributing co-authors were inadvertently omitted from the originally published version of this article. This correction does not affect the scientific content or conclusions of the article. The corrected author list and CRediT Authorship Contributions are included below.

**Claudia D. Fuchs,^1^ Emmanuel D. Dixon,^1^ Philipp Königshofer,^2,3,4^ Thierry Claudel,^1^ Veronika Mlitz,^1^ Marcus Henricsson,^5^ Antonio Molinaro,^5^ Hubert Scharnagl,^6^ Tatjana Stojakovic,^7^ Thomas Reiberger,^2,3,4^ and Michael Trauner^1^**

^1^Hans Popper Laboratory of Molecular Hepatology, Division of Gastroenterology and Hepatology, Department of Medicine III, Medical University of Vienna, Vienna, Austria; ^2^Experimental Hepatic Hemodynamic Lab (HEPEX), Division of Gastroenterology and Hepatology, Department of Medicine III, Medical University of Vienna, Vienna, Austria; ^3^Christian Doppler Laboratory for Portal Hypertension and Liver Fibrosis, Medical University of Vienna, Vienna, Austria; ^4^CeMM Research Center for Molecular Medicine of the Austrian Academy of Sciences, Vienna, Austria; ^5^Department of Molecular and Clinical Medicine, Sahlgrenska Academy, University of Gothenburg, Gothenburg, Sweden; ^6^Clinical Institute of Medical and Chemical Laboratory Diagnostics, Medical University of Graz, Graz, Austria; ^7^Clinical Institute of Medical and Chemical Laboratory Diagnostics, University Hospital Graz, Graz, Austria.

